# Application of python image analysis tools for particle structure detachment detection in high‑speed videos during model filter regeneration

**DOI:** 10.1016/j.mex.2025.103598

**Published:** 2025-08-28

**Authors:** Ole Desens, Jörg Meyer, Achim Dittler

**Affiliations:** Karlsruhe Institute of Technology (KIT), Institute of Mechanical Process Engineering and Mechanics (MVM) – Gas Particle Systems, Straße am Forum 8, 76131 Karlsruhe, Germany

**Keywords:** Particulate matter, High speed imaging, Particulate filters, Python, Detection

## Abstract

Combustion-related particulate emissions are a challenge to air quality and regulatory compliance. In modern combustion engines, wall-flow particulate filters effectively capture soot particles, whereby periodic high-temperature (0_2_) regeneration or passive (NO_2_) regeneration is necessary to reduce the pressure drop. During regeneration, the soot layer breaks up, and small particle structures can detach and be transported further downstream towards the end of the filter channel. A Python-based image analysis workflow is presented for detecting and verifying particle structure detachments in high-speed video recordings of the filter regeneration. The method consists of two integrated modules using OpenCV and NumPy. In the first step, background subtraction (MOG2) and morphological operations are applied to identify candidate structures across video frames. The second step checks the particle structures detected in the first step, isolates a region of interest around the potential detachment and analyzes it using thresholding and pixel-wise difference mapping to confirm or reject the detachment event. Both modules allow parameters to be set and generate visual outputs for verification. The method was validated using a 796,000 frames dataset in which a model filter channel with carbon black loading was regenerated and six small detachment events (*x*_eq_ ≈ 100 - 300 µm) were detected.

• A Python-based method for detection of particle structure detachments in high‑speed videos of model filter regeneration.

• Semi-automated two-step detection and verification of detachments.

• Validated on 796 000 frames, reliably finding detachment events while reducing manual review time.

## Graphical abstract

**Figure:**
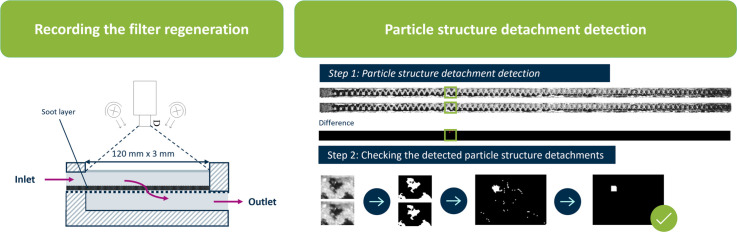

## Specifications table

**Table:** 

**Subject area**	Environmental Science
**More specific subject area**	*Regeneration of wall-flow particulate filter*
**Name of your method**	Particle structure detachment detection
**Name and reference of original method**	*Bradski, G. (2000). The OpenCV Library. Dr. Dobb’s Journal of Software Tools. Retrieved from*https://opencv.org/ *opencv-python contributors. (2025). opencv-python 4.11.0.86: OpenCV bindings for Python [Computer software]. PyPI. Retrieved from*https://pypi.org/project/opencv-python/ *Zivkovic, Z. (2004). Improved adaptive Gaussian mixture model for background subtraction. 17th International Conference on Pattern Recognition (ICPR) (Vol. 2, pp. 28–31). IEEE.*
**Resource availability**	https://github.com/Desens-source/particle-structure-detection.git

## Background

Meeting emission regulations and lowering impact on air quality rely on advanced aftertreatment systems for combustion engines, such as wall‑flow particulate filters (e.g., diesel or gasoline particulate filters), which effectively remove soot particles [1,2]. Over time, the flow through the accumulating particle layer increases pressure drop, necessitating periodic high‑temperature regeneration. During regeneration, the soot layer oxidizes, and the layer breaks up into particle structures that can detach and be transported downstream [[3], [4], [5]]. Filter operation is influenced by the deposited layer: homogeneous layers generally enhance filtration efficiency at the cost of higher pressure drop, whereas plug‑end filling lowers the pressure drop but impairs filtration efficiency [[6], [7], [8], [9], [10]].

Additionally, the soot cake layer in catalytic diesel particulate filters critically influences the temperature distribution and can lead to sharp reaction fronts and local temperature spikes, making catalyst stability crucial [11,12]. Meanwhile, soot distribution inside the catalytic wall enables a more controlled regeneration [11]. Prior investigations show that parameters such as temperature and flow velocity impact layer break-up and particle structure transport [5]. Although optical and microscopic methods enabled the observation of detachment events [3,5], consistently identifying every detachment, particularly smaller or transient structures over the entire channel length remains challenging. *Sappok* et al. [3] *performed visual observation of a diesel particulate filter section during regeneration using optical methods and a stereomicroscope.* To investigate the complete regeneration process, a model filter channel equipped with a quartz glass [5] enables high-speed imaging of the particle layer over the entire channel length during regeneration. Although Thieringer et al. [5] captured detachment events across the full channel length, most studies remain limited to local regions or short time windows, and no automated workflow is known to detect every detachment event during a complete filter regeneration. These high-speed recordings can contain millions of frames, each subject to noise, motion blur, and challenging contrast and lighting conditions. Manual review of such extensive data volumes is labor-intensive and prone to human error. Although widely used, existing powerful particle-tracking software (e.g., TrackMate [13], particle image velocimetry [14]) is not fully optimized for detecting particle structure detachment in a heated channel, where only a handful of particle structures may detach among millions of frames. Detachment events are identifiable between two consecutive frames. Additional challenges include the need to capture small particle structures (100 - 1000 µm) at high temperatures exceeding 500 °C and to minimize false detections caused by image flicker and thermal distortion. These constraints highlight the need for a robust yet flexible method capable of reliably quantifying, locating, and timing particle structure detachments in large video datasets. To address this requirement, a semi-automated Python-based approach has been developed.

## Method details

The approach is implemented across two integrated modules, built on OpenCV [15,16], and NumPy [17], to automatically identify particle structure detachments. The first module detects candidate detachment events by analyzing the entire video frame by frame and the second step is for verifying the candidate detachment events detected in the first module.

### Step 1: particle structure detachment detection

**Fig. 1:**
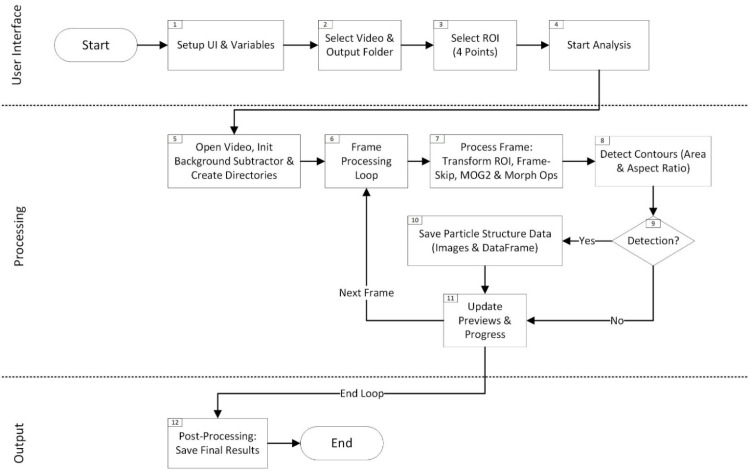
Flowchart of the video‑based particle structure detachment detection method (Step 1).

**Fig. 2:**
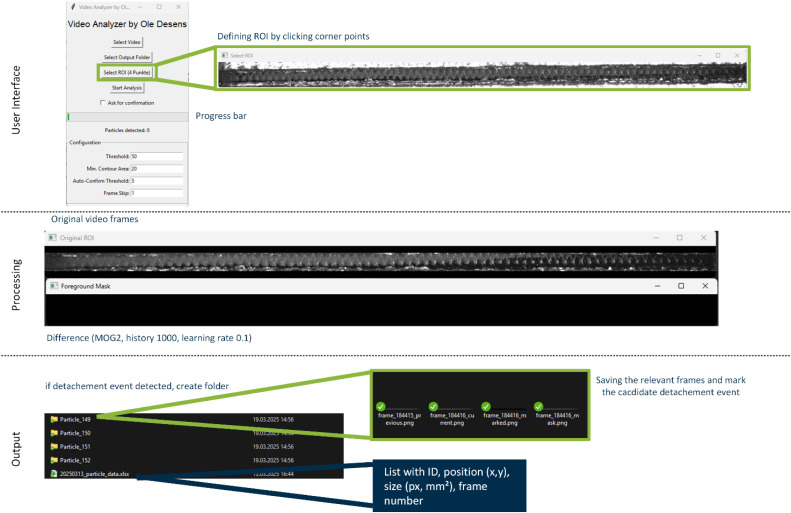
Demonstration of the video‑based particle structure detachment detection method (Step 1) and the resulting output directory containing subfolders that store the relevant video frames and a summary file with particle ID, position, size, and frame numbers.

The first module, as illustrated in the flowchart in Fig. 1 and the demonstration in Fig. 2, provides a graphical interface built using Tkinter (1.1), which prompts the selection of a video file and output directory (1.2). Once a video is selected, the application automatically extracts the first frame to facilitate a region-of-interest (ROI) definition. By clicking four corner points on a displayed preview, the user specifies the exact filter channel boundaries (1.3); these points are then reordered using a dedicated function (order_points) to ensure a consistent arrangement. A perspective transform matrix is calculated from these coordinates, enabling each subsequent frame of the video to be warped into a top-down view of the channel with known physical dimensions (120 mm x 3 mm). After the ROI and calibration parameters (e.g., scaling factors for millimeters per pixel) are confirmed, the code shifts into the main analysis stage (1.4). A separate thread handles video processing to keep the interface responsive. Each incoming video frame is passed through the perspective transform to isolate the ROI. If a specified frame skip is applied, only selected frames are analyzed to reduce computational load in large datasets. The core detection leverages OpenCV’s MOG2 [18] background-subtraction algorithm (1.5), which adaptively learns the channel’s background over a specified history of 1000 frames. A learning rate of 0.1 integrates each new frame into the model at a 10 % weighting, gradually updating the background estimate as conditions change (1.7). Morphological opening (Kernel size = 3,3; iterations = 2) and closing (iterations = 1) operations help suppress noise and small artifacts, while cv2.findContours identifies candidate particles that exceed user-defined size (e.g. 20 px) and aspect-ratio thresholds (e.g. 1:4) (1.8, 1.9). Whenever a new contour is confirmed, the code saves four reference images (previous frame, current frame, marked contour overlay, and filled-mask image) into a dedicated particle subfolder (1.10). The contour area is calculated in pixels and converted to real-world units using the defined scale factors. Intermediate results are logged in a pandas DataFrame and periodically written to a temporary .CSV file to prevent data loss over long runs. Once all frames have been processed, the final dataset is exported as an Excel file, and any temporary files are consolidated or removed (1.12). By reviewing the list and the images, the user can verify the accuracy of each identified detachment event. If any detection proves incorrect, the user can discard or reclassify it.

### Step 2: checking the detected particle structure detachments

**Fig. 3:**
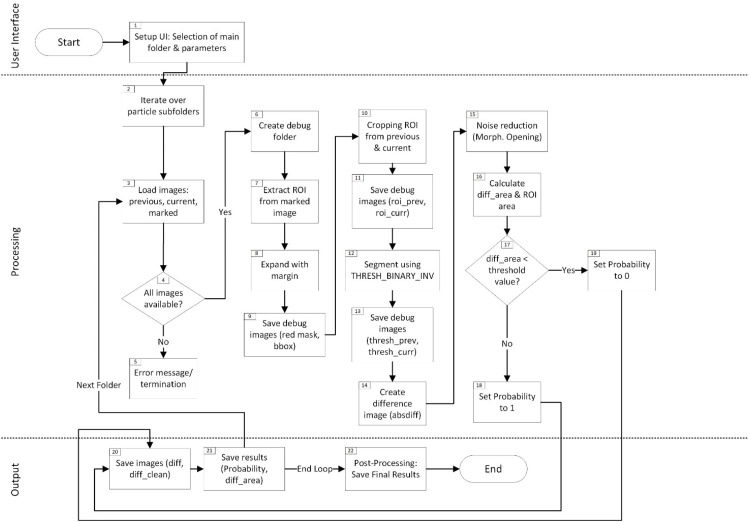
Flowchart illustrating the verification process (Step 2) on the left, where each detected particle folder is analyzed by extracting the ROI from the marked image, segmenting previous vs. current frames, and computing a difference image to assign a detachment probability.

**Fig. 4:**
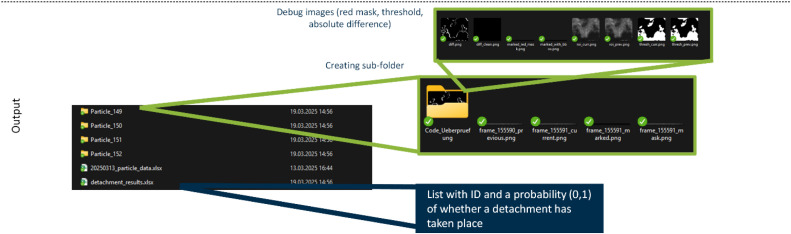
Demonstration illustrating the output of the verification process (Step 2).

In the second step shown in Fig. 3, the script focusses on the relevant sub-region within each saved image from step 1 by reading the marked image file (2.3), which highlights the supposed detachment area in red. Through an HSV-based masking step, the red contour is isolated and used to determine a bounding box that covers only the presumed detachment region (2.7). By cropping both the previous and current images to this bounding box while adding a small user-defined margin (2.8–2.10). Unwanted background from the rest of the filter and extraneous details are excluded. This targeted examination significantly refines detection compared to Step 1, as it no longer scans the entire video frames (where minor fluctuations can trigger false detections). Following the region extraction, the code uses THRESH_BINARY_INV to binarize the cropped images (2.12), turning darker particle structures white and lighter background black (2.12). An absolute difference image of the two thresholded frames highlights changes in the particle’s shape or position (2.14). Morphological opening (Kernel size = 3,3; iterations = 1) then reduces noise and small artifacts (2.15). If the area of white pixels in this difference image exceeds a threshold of 20 px, the script labels the detachment as correct (probability = 1) (2.19). Otherwise, the detection is flagged as incorrect (probability = 0) (2.18). The minimum area threshold is set to 20 px in both modules, corresponding to an area-equivalent diameter of roughly 100 µm. This choice aligns with commonly referenced visibility limits, since unaided human vision typically can discern features in the 100 µm range at normal viewing distances [19]. Because this procedure strictly confines its calculations to the region outlined in the marked image, potential distractions such as channel edges, random flicker, or lighting shifts in irrelevant parts of the frame are excluded from the analysis. Consequently, the likelihood of identifying truly detached particle structures is improved, while false candidates from Step 1 are reduced. As a result, the second script generates a set of debug images (see Fig. 4), stored in a subfolder within each particle’s directory, which document the cropped region (2.9, 2.11), the binary threshold (2.13), and the difference mask (2.20). These images allow a quick visual check to confirm whether the flagged candidate detachment is correct or not. Additionally, the script compiles all probability values set either to 0 (no meaningful change detected) or 1 (significant difference detected) into an Excel file (2.21, 2.22), along with the measured difference area in pixels. This output serves as a record of which candidate detachment events from Step 1 were validated or invalidated, helping to identify common sources of false candidates and refine future detection parameters. Consequently, the entire workflow not only locates possible detachment events but also provides a methodical way to verify them.

## Method validation

A representative test was conducted using a model filter channel loaded with carbon black, where a quartz glass window was integrated into the channel wall to allow perpendicular high‑speed imaging. Once the carbon black layer began visibly breaking up, a CP90‑25P‑M72 camera (Optronis GmbH) recorded 796,000 frames at 1000 fps under 150 W LED panel illumination (GSVitec MultiLed G8). The camera, equipped with a Zeiss Macro lens (Milvus 2/100 M), captured images at 5120 × 200 px with a 1/10,000 s exposure time. Table 1 summarizes the key parameters for this dataset, which included only six small particle structure detachments (x_eq_ ≈ 100 - 300 µm), precisely why it was selected to test the method’s capability for detecting subtle events.

**Table 1:** Test dataset.

Total number of frames	796,000
Recording speed	1000 fps
Particle structure detachments	6

**Fig. 5:**
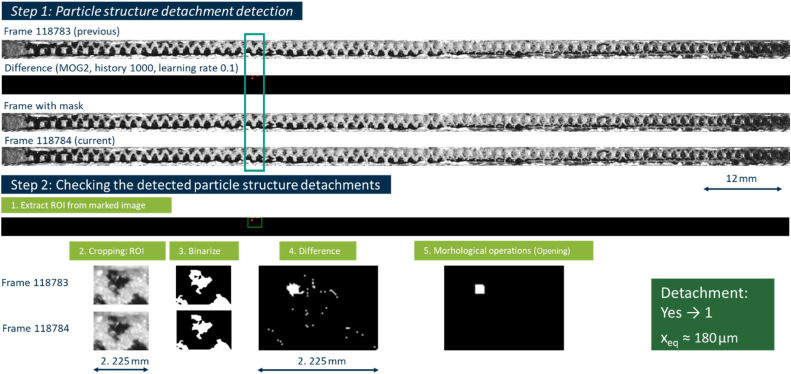
Example of a correctly detected detachment.

**Fig. 6:**
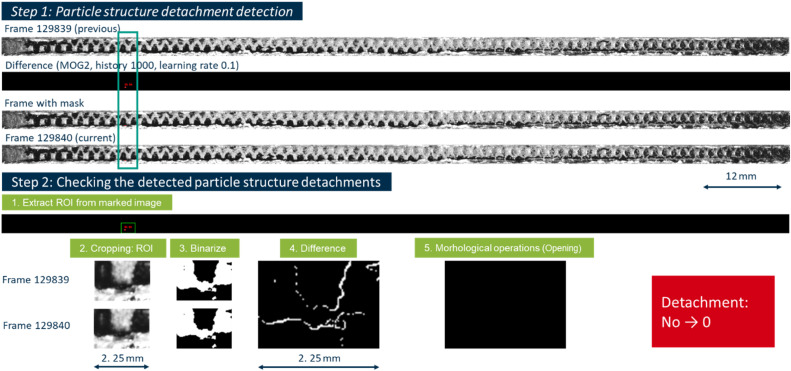
Example of an incorrectly detected detachment.

Using the first module in step 1, 452 potential detachment events were detected. An example of correct and incorrect detections are shown in Fig. 5, Fig. 6, where both scripts run in sequence to locate and verify potential particle structure detachments.

These examples present the saved frames in which a potential particle structure detaches from the filter surface. In Step 1, the MOG2 [18] background subtraction algorithm flags a new contour, which is outlined in red. The script creates a mask and saves both the current and previous frames in a “Particle” folder. In Step 2, only the region outlined by the red contour is extracted from both frames. A binary threshold (THRESH_BINARY_INV) reveals the particle structure as a white shape, and an absolute difference image confirms that a distinct area of the particle structure has moved. Because this change exceeds the minimum area threshold, the method classifies the event as a valid detachment. The area-equivalent particle diameter of these particle structure is approximately 180 µm and is so small that this detachment would be easy to miss without this methodology. Fig. 6 shows again a highlighted potential detachment contour from Step 1. After cropping the region in Step 2 and applying the threshold, building a difference image and applying an opening operation, the script finds that the actual change in pixel area does not surpass the specified threshold of 20 px, the detected region is primarily due to minor frame-to-frame changes, such as flicker by thermal noise, rather than a correct particle structure detachment. The resulting debug image is black, the event is consequently labeled an incorrect detection.

Table 2 summarizes the detection results obtained from the video dataset comprising 796,000 frames. In the first step, 452 structures were flagged, although only 6 were correct detachments yielding a rejection rate of 98.7 %. The second step targeted ROI extraction, thresholding, and difference mapping, reducing the total detections to 8 and misclassifications to 2, thus correctly identifying all 6 detachments with a 25.0 % rejection rate. This two-stage approach thereby significantly decreases false candidates compared to simpler difference methods without morphological filtering or MOG2, which can exceed >>10,000 candidate detachment events in 300,000 frames (5 min video data) if parameters are not optimized. Importantly, storing all relevant images in “Particle” subfolders allows to manually confirm or reject cases, ensuring that even subtle detachment events are found while minimizing excessive candidate detections. The workflow thus achieves its key goal: detecting all real detachment events while markedly reducing the manual verification effort.

**Table 2:** Results from the test dataset with 796,000 frames.

	Candidate detachment events	Correctly detected detachment events	Rejection rate
Step 1	452	6	98.7 %
Step 2	8	6	25.0 %

For future improvements, deep-learning based segmentation models alongside the current MOG2/morphological pipeline may enhance robustness against variable lighting and thermal distortion. This method is suited to future comparative studies of filter regeneration in a model channel under varied conditions such as different soot types, operating parameters (flow velocity, temperature), regeneration atmospheres (O₂ vs. NO₂), and catalyst formulations to systematically map how these factors influence detachment behavior of soot particle structures.

## Limitations

This method’s parameters (e.g., contour area threshold, morphological settings) may need iterative tuning for different video conditions. When the camera or magnification is changed, the contour-area threshold and morphological kernel parameters must be scaled according to the new pixel-to-mm calibration. If the recording frame rate varies, the MOG2 history length should be adjusted proportionally to the actual fps. Swapping filter substrates (e.g. cordierite, SiC or fiber monoliths) may require fine-tuning the intensity threshold to accurately separate particle structures detachments from the background. These adjustments can be made iteratively in a small representative video section using the debug images. Poor contrast, significant motion blur, or strong thermal shimmer can increase candidate detections. Manual checks of debug images are still required to confirm small detachments and rule out artifacts.

## Ethics statements

Not applicable.

## Author's Role

**Ole Desens:** Conceptualization, Methodology, Software, Validity tests, Data curation, *Visualization*, Writing- Original draft preparation*, Project administration.* ***Jörg Meyer:*** Conceptualization, *Writing- Reviewing and Editing.* ***Achim Dittler****:* Conceptualization, *Supervision, Writing- Reviewing and Editing, Funding acquisition.*

## Declaration of competing interest

The authors declare that they have no known competing financial interests or personal relationships that could have appeared to influence the work reported in this paper.

## Data Availability

Data will be made available on request.
